# Elevated Incidences of Antimicrobial Resistance and Multidrug Resistance in the Maumee River (Ohio, USA), a Major Tributary of Lake Erie

**DOI:** 10.3390/microorganisms9050911

**Published:** 2021-04-24

**Authors:** Maitreyee Mukherjee, Leah Marie, Cheyenne Liles, Nadia Mustafa, George Bullerjahn, Terry J. Gentry, John P. Brooks

**Affiliations:** 1 School of Biological, Environmental, and Earth Sciences, The University of Southern Mississippi—Gulf Park Campus, 730 East Beach Blvd, Long Beach, MS 39560, USA; 2 Department of Soil and Crop Sciences, Texas A&M University, College Station, TX 77843, USA; leahkay4@tamu.edu (L.M.); cheyenne.liles@gmail.com (C.L.); tjgentry@tamu.edu (T.J.G.); 3 Department of Biological Sciences, Bowling Green State University, Bowling Green, OH 43404, USA; nadiam@bgsu.edu (N.M.); bullerj@bgsu.edu (G.B.); 4 USDA-ARS, Mississippi State, MS 39762, USA; john.brooks@usda.gov

**Keywords:** *E. coli*, antimicrobial resistance, antibiotics, watershed, multidrug resistance

## Abstract

Maumee River, the major tributary in the western basin of Lake Erie, serves as one of major sources of freshwater in the area, supplying potable, recreational, and industrial water. In this study we collected water samples from four sites in the Maumee River Bay between 2016–2017 and *E. coli* was isolated, enumerated, and analyzed for antimicrobial resistance (AMR) and multidrug resistance (MDR). Strikingly, 95% of the total isolates were found to be resistant to at least one antibiotic. A very high resistance to the drugs cephalothin (95.3%), ampicillin (38.3%), tetracycline (8.8%), gentamicin (8.2%), ciprofloxacin (4.2%), cefoperazone (4%), and sulfamethoxazole (1.5%) was observed within isolates from all four sampling sites. Percentages of AMR and MDR was consistently very high in the summer and fall months, whereas it was observed to be lowest in the winter. A remarkably high number of the isolates were detected to be MDR—95% resistant to ≥1 antibiotic, 43% resistant to ≥2 antibiotics, 15% resistant to ≥3 antibiotics, 4.9% resistant to ≥4 antibiotic and 1.2% resistant to ≥5 antibiotics. This data will serve in better understanding the environmental occurrence and dissemination of AMR/MDR in the area and assist in improving and establishing control measures.

## 1. Introduction

Bacteria are proficient in developing antimicrobial resistance (AMR) when exposed to antibiotics through processes such as horizontal gene transfer between related or non-related species [1,2], or by genetic transfer mechanisms of transformations, transduction, or conjugation [2,3,4]. Use of antibiotics by humans over decades in clinical and pharmaceutical environments for treatment of diseases and in agricultural environments, through inappropriate use in farm animals, has led to rapid development of AMR worldwide—a critical global threat identified by several health organizations such as the World Health Organization, U.S. Centers for Disease Control, the National Academy of Science’s Institute of Medicine, the Federal Interagency Task Force on Antimicrobial Resistance, the Infectious Diseases Society of America and numerous other worldwide public health authorities [5,6,7,8]. Over decades, clinical deployment of new antibiotics has quickly been followed by the evolution of bacteria able to resist their effects [2]. Furthermore, bacteria may acquire AMR to multiple antibiotic classes, leading to increased presence of multidrug resistance (MDR) in bacterial populations, particularly potential pathogens, further adding to the complexity of this life-threatening global issue [9].

Emergence of AMR and MDR bacteria coupled with a dearth of novel antibiotics has therefore led to several complex public health issues including increased hospital stays, lack of treatment options, and increased mortality and morbidity rates [10,11,12,13,14,15,16,17,18,19]. It is critically important from public health perspectives to track the sources of AMR and MDR bacteria in order to implement proper management strategies to control their occurrence and distribution. Although considerable research in the past several decades have focused on studying the prevalence of AMR and MDR in clinical and farm environments, comparatively little work has been done so far to examine the distribution of these organisms through natural and artificial environmental reservoirs [7,20,21]. In addition, the role of urban wastewater treatment plants as a highly favorable setting for facilitating development of AMR and MDR within bacterial populations and their subsequent release into the environment through direct effluents has also been pointed out in the recent years [11,21,22,23,24].

*Escherichia coli*, naturally present in the human and animal intestinal tract, is usually disseminated into the environment through fecal sources and has been found to grow and survive in natural environments including water, soil and sediment [25]. Sources of *E. coli* contamination in watersheds can include humans [26], farm animals [27], pets [28] and wildlife [29,30]. Therefore *E. coli* is widely used as an indicator for fecal contamination in watersheds [31], and can be used to examine AMR and MDR in many different natural and managed environments including soil and water sources [30,32,33,34,35,36,37,38].

The Maumee River is the key tributary in the western basin of Lake Erie. The river is a major source of fresh water—potable, recreational, and industrial water—in the area. The river runs through a primarily agricultural watershed, ultimately flowing through the largely urban city of Toledo and emptying into the western basin of Lake Erie. Due to annual occurrences of harmful algal blooms in the river and Lake Erie driven by agriculturally derived nutrients, several studies thus far have focused on studying the algal blooms in the area (reviewed in Bullerjahn et al. [39]). The extent of nutrient pollution had led to a recommended 40% reduction in nutrient loads in order to mitigate the Lake Erie blooms [40]. However, no study thus far has examined the presence of coliform bacteria, and the distribution and occurrence of AMR and MDR in the watershed. This is particularly important considering the river is a source of drinking water for western Lucas County and Wood County, OH, and the river is a source for recreation (boating and fishing) from spring through fall. Notably, with respect to coliforms of possible animal origin, the Maumee River watershed has seen a 42% increase in animal feeding operations since 2005, resulting in an increase in manure in the region from 3.9 million tons to 5.5 million tons per year during this period [41,42]. Additionally, our sampling sites are adjacent to the wastewater treatment plant serving urbanized western Lucas County (OH) upstream from Toledo. Studying the occurrence of AMR and MDR in the area is warranted because of the likely implications it has on public health, agricultural, industrial, and environmental regulations that must be considered in concert with management decisions regarding nutrient loadings. This study documents the potential threat to human health due to AMR/MDR, setting the stage for future monitoring and remediation efforts distinct from, but complementary to, the nutrient management plans currently being implemented [40].

In this study we collected water samples from four different sites in the Maumee River between 2016–2017. From these samples, a total of 329 *E. coli* isolates were analyzed for AMR and MDR by antimicrobial sensitivity testing with a goal to better understand the incidence and manifestation of AMR and MDR in the area.

## 2. Materials and Methods

### 2.1. Sampling Sites, Study Duration and Sampling Methods

**Figure 1:**
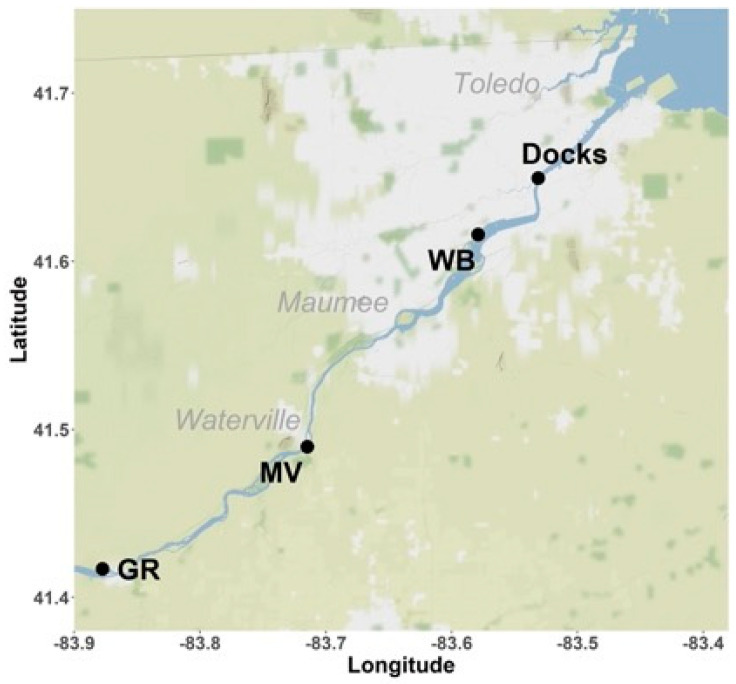
Map of the Maumee river watershed showing the four sampling sites. Lake Erie is seen here on the top right corner where the Maumee river drains into the lake. Site identifier as follows: GR: Grand Rapids; MV: Miltonville; WB: Walbridge Park.

Four sites were chosen, beginning upstream of Lake Erie. The sites sampled, upstream to downstream are Grand Rapids (GR; 41.416944, −83.8775), Miltonville (MV; 41.48972, −83.71472), Walbridge Park (WB; 41.61583, −83.57889) and the Docks (41.64944, −83.5311) (Figure 1). The Grand Rapids site is in a rural area and receives runoff from agricultural land. Miltonville is a suburban site directly across the river from the town of Waterville, OH and the Lucas County Wastewater Treatment Plant (LC-WWTP). The Walbridge Park and Docks sites are both located within the city of Toledo, OH and are affected by urban stormwater runoff and industrial inputs. The Docks site lies 3 km upstream from the mouth of the river at Lake Erie. At the sites, water temperatures ranged between 1 °C (December 2017) and 4 °C (November 2017) in the cooler months (USGS Gage 04193490, Waterville, OH, USA). During the warmer months, the daytime river temperature in May 2017 was 15 °C, and the June and July temperatures were 22 °C and 24 °C, respectively (USGS Gage 04193490). Surface water samples were collected in sterile polycarbonate bottles from each of the four sites described above in 2016 (1st October, 12th November, 3rd December) and 2017 (25th May, 6th and 14th June, 6th and 24th July). Due to extreme cold conditions and partial freezing of the river during winter months, sampling was paused during winter. Samples were transported to the Bowling Green State University (Bowling Green, OH, USA) lab under ice, and immediately processed for *E. coli* enumeration and isolation.

### 2.2. E. coli Strains Archival and Enumeration 

*E. coli* strains were isolated and enumerated from collected water samples using the USEPA 1603 method (USEPA, 2005). *E. coli* colonies were isolated on modified mTEC agar plates from each water sample using dilution and filtering method with a 0.45 µm grid filter. The purple colonies appearing on the mTEC agar plates were then enumerated and 2–3 colonies randomly selected and streaked onto nutrient MUG (4-methylumbelliferyl-β-D-glucuronide) agar plates for verification. Ten mL liquid cultures were prepared in LB medium prior to the addition of glycerol to 50%. Glycerol stocks were archived at −80 °C for future study. A total of 329 *E. coli* isolates were prepared for AMR and MDR testing at Soil and Aquatic Microbiology Laboratory (SAML) at Texas A&M University (College Station, TX, USA).

### 2.3. Antimicrobial Resitance Analysis

The Kirby Bauer disk diffusion [43] method was used to test for antimicrobial resistance in each sample. First, each isolate was streaked onto tryptose soy agar (TSA) and incubated at 35 °C for 18–24 h to obtain fresh colonies from each isolate. After this, individual isolated colonies were inoculated into tryptose soy broth (TSB) and incubated at 37 °C with continuous shaking at 150 rpm in a temperature-controlled incubator. After 3 h, or until the pure cultures obtained a turbidity of at least 0.5 McFarland’s standard (approximate concentration of 10^7^–10^8^ cfu ml^−1^), the suspension(s) was spread onto Mueller Hinton Agar with sterile cotton swabs. Antibiotic disks were then dispensed onto the agar plates using an automatic sterile disk dispenser (BBL^®^ Self-Tamping Sensi-Disc™ Dispenser, Franklin Lakes, NJ, USA). The plates were incubated at 37 °C overnight for the appearance of zones of inhibition. Each isolate was tested for resistance to the following antibiotics—tetracycline (TE-30/disk potency-30 µg), ampicillin (AM-10/10 µg), cephalothin (CF-30/30 µg), cefoperazone (CFP-75/75 µg), imipenem (IPM-10/10 µg), gentamicin (GM-120/120 µg), sulfamethoxazole (SXT/23.75 µg), and ciprofloxacin (CIP-5/5 µg). These antibiotics were chosen to represent the majority of classes of antimicrobials, as well as for their known efficacy against wildtype *E. coli*. Additionally, these drugs include classes of antimicrobials which have been used both in agriculture (tetracyclines, cephalosporins, sulfamethoxazole, ciprofloxacin) as well as in patient care. The three control reference strains used with each testing event were *Pseudomonas aeruginosa* (ATCC 27853), *Staphylococcus aureus* (ATCC 25923), and *E. coli* (ATCC 25922). Once images of each tested agar plates were obtained (UVP GelDoc-It imaging system, Analytik Gena US LLC, Upland, CA, USA), the zone diameter measurements were taken using the software ImageJ [44]. Inhibition zone diameters were measured and recorded in millimeters and compared to Clinical and Laboratory Standards Institute (CLSI) standards to determine if each isolate was susceptible or resistant to each antibiotic. Each isolate was then recorded for their observed resistance to multiple antibiotics. To analyze MDR, binomial resistance values of each isolate were determined and grouped into five categories of resistance to ≥1, ≥2, ≥3, ≥4 and ≥5 antibiotic categories.

### 2.4. Statistical Analysis

A one-way ANOVA was utilized to compare *E. coli* mean levels between sampled site and sample month, respectively. Means were considered significantly different at *p* < 0.05. Prior to analyses, all *E. coli* means were log10 transformed to achieve normal distribution. All pairwise means were post-hoc compared utilizing Fisher’s least square difference. A Chi square test for independence was applied to test significant differences between *E. coli* antibiotic susceptible and resistant proportions based on sampled sites and sample month as independent variables, respectively. To avoid asymptomatic cells, isolates were binned to resistant or susceptible, and MDR ≥2 or ≥4 categories, respectively. In addition, collection month was binned to Fall/Winter and Spring/Summer seasons; however, no significant differences were noted between binned seasons and will not be further discussed. Any tested relationship was considered to be significant at *p* < 0.05, or when the Chi square sum was greater than 3.84. Post-hoc multi-comparison tests were carried out, where appropriate, by conducting pairwise Chi square tests for sample month and Bonferroni correction.

## 3. Results

### 3.1. E. coli Population

**Figure 2:**
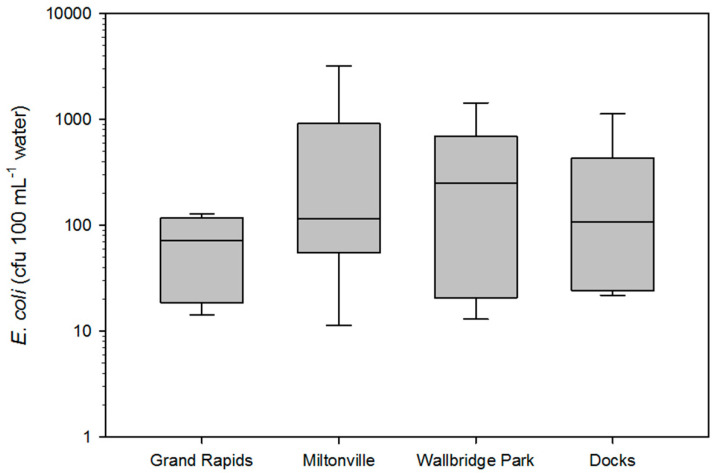
Box plot displaying *E. coli* cfu 100 mL^−1^ of water sample within the four sites across all sampling events. The line within each box indicates the median value. The whiskers above and below the box indicate the maximum and minimum values of *E. coli* load within the four sites.

Across all sampling events, Miltonville, the site next to the LC-WWTP displayed the highest mean *E. coli* cfu 100 mL^−1^ (526 cfu 100 mL^−1^ water), while Grand Rapids, the site most upstream from Lake Erie displayed the lowest mean *E. coli* cfu 100 mL^−1^ (60.76 cfu 100 mL^−1^ water) (Figure 2). Walbridge Park, the site within the metropolitan and industrial district of the city of Toledo displayed the second highest prevalence of *E. coli* (mean 425 cfu 100 mL^−1^ water). Overall, there was no significant difference found between mean *E. coli* levels when means were compared by site (*p* > 0.05). Of all the sampling events, the *E. coli* levels fell below the water quality standards of 126 cfu 100 mL^−1^ set by EPA and the Ohio-EPA at least 50% of the times (Table 1) (EPA, 2012, 820-F-12-058; OH EPA 3745-1-37). Sampling month was significantly associated with differences in mean *E. coli* levels (*p* < 0.05); particularly, means associated with October 2016, December 2016, and July 2017 which were significantly greater than means associated with other months (*p* < 0.05). Between the sampling months, the month of October 2016 recorded the highest coliform numbers, likely because of the warmer climate in the watershed around that time.

### 3.2. Antimicrobial Resistance Patterns

Overall, across all sites and sampling dates, highest resistance was observed to the drugs cephalothin (95.3%), followed by ampicillin (38.3%), tetracycline (8.8%), gentamicin (8.2%), ciprofloxacin (4.2%), cefoperazone (4%), and sulfamethoxazole (1.5%) (Table 2). Of all antibiotics, cephalothin resistance was observed to be highest (*p* < 0.05) across all sampling events and all sites (85.5–95.3%) (Table 2). Resistance rates associated with ampicillin and tetracycline, when compared with all other antibiotics tested in pairwise comparisons, were found to be greater (*p* < 0.05), except tetracycline compared with gentamicin, which was not statistically different (*p* > 0.05). Rates of resistance to ciprofloxacin and cefoperazone, were not statistically different (*p* > 0.05) but were greater than sulfamethoxazole resistance rates (*p* < 0.05). Resistance to imipenem and sulfamethoxazole were not statistically different (*p* > 0.05); while, resistance to gentamicin was statistically greater than that of sulfamethoxazole (*p* < 0.05). Resistance rates to imipenem and tetracycline were also statistically different (*p* < 0.05).

**Figure 3:**
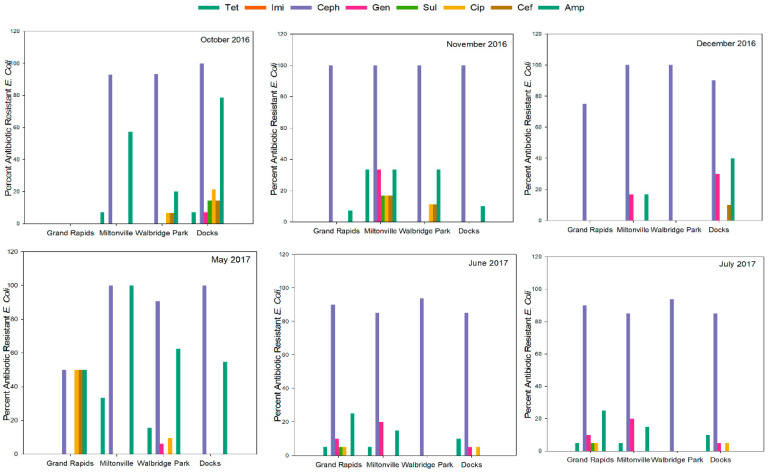
Percentage of antimicrobial resistance (AMR) within *E. coli* isolates in the four sites across all sampling events. Tet—Tetracycline, Ceph—Cephalothin, Sul—Sulfamethoxazole, Cef—Cefoperazone, Amp—Ampicillin, Cip—Ciprofloxacin, Gen—Gentamicin, Imi—Imipenem.

Site did not exert any significant effect on antibiotic susceptibility rates (*p* > 0.05). Except for cephalothin, resistance of isolates to all other antibiotics was observed to be higher in the downstream sites when compared to the upstream ones, though not significantly. This may be because of the cumulative effect of agricultural, industrial, urban and added storm water runoff into the downstream sites of the river bay. Across all the months sampled, the percentage of AMR was found to be consistently greater in the warmer months of summer and fall (October and November 2016, May, June, and July 2017), whereas it was observed to be lowest in the colder month of December 2016 (Figure 3). 

However, most antibiotics were not significantly influenced by sample month. Tetracycline and ampicillin resistance rates were the only antibiotics tested which were influenced by sample month (*p* < 0.003); however, upon post-hoc pairwise comparisons of rates, only ampicillin resistance was found to be more prevalent in May and June compared with December. Similarly, ampicillin resistance rates from June samples were more prevalent than July (*p* < 0.003). Of all, only one isolate from Docks in June 2017 was found to be resistant to the antibiotic Imipenem, belonging to the last-resort Carbapenem category of antibiotics.

### 3.3. Multidrug Resistance Patterns

**Figure 4:**
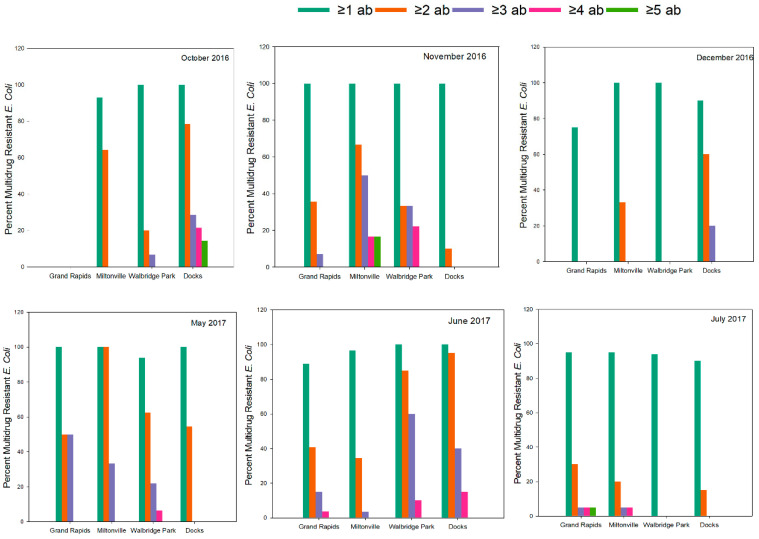
Percentage of multidrug resistance (MDR) within *E. coli* isolates in the four sites across all sampling events.

One of the most striking findings in this study was that 313 out of the 329 *E. coli* isolates (95%) was found to be resistant to at least one antibiotic (Table 3), primarily associated with cephalothin and ampicillin, two β-lactam drugs. Presence of MDR (resistance to ≥2 antibiotics) was also noted to be high in all sites tested—144 out of the 329 (43%) of the isolates belonging to the ≥2 antibiotics category and 50 isolates (15%) belonging to the ≥3 antibiotics category. As stated, β-lactam drugs drove the majority of the MDR. On an average across all sampling events (including the colder months), the highest MDR incidence was observed in the downstream sites compared to the upstream sites: Walbridge Park (44.3% isolates resistant to ≥2 antibiotics) and Docks (54.1% isolates resistant to ≥2 antibiotics) in contrast to Grand Rapids (33.3% isolates resistant to ≥2 antibiotics) and Miltonville (41% isolates resistant to ≥2 antibiotics). Of these, 6.2% from Walbridge Park and 6% from Docks were found to resistant to ≥4 different antibiotics across all sampling events (Table 3). However, site had no statistical influence on MDR to ≥2 or ≥4 antibiotics (*p* > 0.05). Interestingly, sample month had little influence on MDR to ≥2 or ≥4 antibiotics. Post-hoc pairwise comparisons of MDR to ≥2 antibiotics showed that MDR isolates only from July were statistically different from those collected in June and Oct., respectively (*p* < 0.001). Except for the colder months of December, we found isolates that are resistant to ≥4 antimicrobial agents in all other months, mostly in the three downstream sampling sites of Miltonville, Walbridge Park and Docks (Figure 4). Notably, 22.2% (November ’16), 6.2% (May ’17) and 10% (June ’17) of isolates from Walbridge Park fell into the ≥4 category. Within the Docks site 21.4% (October ’16) and 15% (June ’17) were resistant to ≥4 antibiotics. Within the Miltonville site, at least 16.6% (November ’16) and 5% (July ’17) belonged to the ≥4 MDR categories (Figure 4), though not statistically significant, owing to overall lower MDR prevalence.

## 4. Discussion

In the Maumee river watershed, research has been largely focused on the major role the river plays in draining upstream agricultural lands that have fueled toxic cyanobacterial blooms in Lake Erie. The sampling sites reflect the partitioning of land use between agricultural drainage and urbanized areas downstream, and these data indicate that coliforms are commonly detected at all sites. Occurrence of antimicrobial resistance and multidrug resistance as observed across the sampling sites and months within this watershed is of great concern, considering the importance of role of the Maumee River Bay in the area due to its role in the human, agricultural and industrial practices in the area.

The observation that the percentage of AMR/MDR was greater in the summer months could be explained by differences in source contributions throughout the year. For instance, during the November and December 2017 sampling dates, agricultural contributions would likely be lower due to the onset of winter. By Ohio law, Senate Bill 1 of the 131st Assembly states that manure cannot be spread onto frozen ground in an effort to minimize runoff of nutrients into the watershed.

The highest resistance among all isolates to the drug Cephalothin (90–100%) is not surprising as these results are consistent with previous findings from other studies [34,35,42,43]. Sayah et al. [35] found very high cephalosporin (e.g., cephalothin) resistance (as high as 80.6%) in *E. coli* isolates—in surface water samples in the Red Cedar watershed in central Michigan. A similar study conducted in the urbanizing Carter’s Creek watershed in central Texas [45] also reported high cephalothin resistance (84%) in surface water samples. Similarly, Janezic et al. [46] also reported high cephalothin resistance in their study (80%) from several locations in Illinois and Missouri. Ampicillin resistance was observed to be the 2nd highest. It is worth mentioning here that both these antibiotics are beta lactams: ampicillin is a 3rd generation penicillin, while cephalothin belongs to 1st generation cephalosporins. Both these antibiotics target penicillin binding proteins (PBP), thus resistance to either could be possibly related based on the relative observation of their patterns of resistance. Furthermore, Mukherjee et al. [30] has also observed similar outcomes in a study on rates of AMR/MDR in soil and runoff water samples collected from three experimental watersheds in Riesel, TX.

Tetracycline resistance in our water isolates was also comparable to previous studies. Sayah et al. [35] observed a 27.3% tetracycline resistance, whereas another study in the Carter’s Creek watershed in Texas [47] also found a substantial occurrence of tetracycline resistant genes in both sediment and surface water samples. Ampicillin resistance found in the Maumee Bay watershed in our study was considerably higher in some of our sites than several other studies. For example, Castillo et al. [34] reported a higher rate of ampicillin resistance in the San Pedro river watershed (39.3%), Ibekwe et al. [32] reported ~5% ampicillin resistance in most of their surface water sites, whereas Laird (2016) found ampicillin resistance to be between 5–28% in the Carter’s Creek watershed.

Sulfamethoxazole resistance detected in this study was also within the range of detection compared to that observed in several other studies. Sayah et al. [35] observed an overall 2.4% sulfamethoxazole resistance, whereas Ibekwe et al. [32] detected a higher resistance (39% in surface water), and Servais et al. [36] reported an overall 16% resistance to sulfamethoxazole in a Seine river watershed.

Guyomard-Rabenirina et al. [48] reported that 11.8% of their isolates from the Guadeloupe River watershed were resistant to the antibiotic gentamicin. Less than 1% of 462 *E. coli* isolates were found to be gentamicin resistant in the study conducted by Edge et al. [37] in Hamilton, Ontario region. Furthermore, previous studies [46,49] have also reported incidence of <1% gentamicin resistance is surface water. Overall resistance observed within our isolates to gentamicin was comparably on the higher side, and is of concern since gentamicin is currently a frequently used antibiotic for treatment of infectious diseases.

Resistance to imipenem, a group 2 carbapenem, is usually considered the last line of defense against Gram-negative pathogens [50]. Carbapenem is therefore used extremely cautiously in treatment of infectious diseases. The occurrence, spread, and distribution of carbapenem-resistant enterobacteriaceae (CRE) is of grave concern to public health because of the limited treatment options and high mortality rates associated with CRE infections [51]. In our study we found at least one isolate that was carbapenem resistant, which was also resistant to at least two other antibiotics. While, relatively low in occurrence, the presence of carbapenem resistant enterobactericae in the environment is concerning, however this corroborates other studies [52].

Urban wastewater treatment plants (WWTPs) are increasingly being suspected to be one of the major reservoirs of ARGs and their release into the environment through effluents [11,21,22,23,24,53,54,55,56,57,58]. Laird et al. [46] and Mukherjee et al. (unpublished data) in their study on an urbanizing watershed in College Station, TX also found very high occurrences of AMR and MDR *E. coli* specifically highest within the sites that were immediately next to a WWTP. Although not conclusive, these observations are consistent with our findings in this study, where we found a high incidence of AMR on several instances near the Lucas County WWTP (Miltonville).

It is important to note that only selected antibiotics were tested in this analysis—further insights could be obtained into these interpretations with a more extensive antibiotic ‘resistome’ study. Since *E. coli* is considered an indicator of potential fecal pathogens, these results may be indicative of resistance profiles associated with other enterobactericae, however, bacterial species are subject to acquiring resistance genes at different rates and with different mechanisms, thus the current results require further investigations. Furthermore, it is also important to mention here that the study was limited by culture-based methodologies, and additional investigations targeting antibiotic resistant genes in these sites may reveal further understanding of the interrelationships between the AMR/MDR bacterial strains, antibiotic resistant genes, antibiotic agents, specific microbial species, and environmental sources of AMR dissemination to help to eventually enable the modeling of antibiotic resistance transfer through the environment.

## 5. Conclusions

This is the first study identifying the incidences of AMR and MDR in the Maumee River watershed. The study finds extremely high incidences of AMR and MDR within the watershed. Of the 329 isolates, 313 (95%) were found to be resistant to at least one antibiotic. In addition, a high number of the isolates were found to be resistant to ≥2 antibiotics (43%), ≥3 antibiotics (15%), ≥4 antibiotics (4.9%), and ≥5 antibiotics (1.2%). We also noted a cumulative increase in MDR values in the downstream sites of the watershed (Walbridge Park and Docks) located within the more industrial and metropolitan district of the city of Toledo. These results indicate a possible urban and industrial influence on the occurrence and distribution of AMR and MDR in the area. In addition, the study also finds a high resistance to the antibiotics cephalothin (95%), ampicillin (38%), tetracycline (8.8%) and gentamicin (8.2%), ciprofloxacin (4.2%), cefoperazone (4%), and sulfamethoxazole (1.5%). This is of great concern as many of these antibiotic groups are still used as primary antibiotics in clinical treatment procedures. According to the Centers for Disease Control (CDC)’s latest antibiotic resistance report (CDC, 2019), it is vital for scientists and agencies to track the occurrence and distribution of AMR/MDR in the environment to improve the gaps in our understanding of the environmental dissemination and spread of AMR/MDR in order to develop control measures for this most pressing health issue worldwide. Currently, the Maumee River is studied almost exclusively as a source of nutrients that fuel harmful algal blooms in Lake Erie. Consequently, all efforts in managing the river and watershed are focused on the reduction of agriculturally derived phosphorus and nitrogen. This paper is the first to document that the river poses potential threats to human health through exposure to drug-resistant commensals and pathogens. Given the detection of AMR and MDR isolates in the river, efforts should also focus on limiting combined sewer overflows and runoff of animal waste from the upstream agricultural landscape. Since the Maumee River serves as one of the most important water sources in this area, it is vital to study how AMR/MDR may spread and disseminate within this specific watershed, as this data will be beneficial in establishing/modifying future environmental, agricultural, and industrial regulations in the area.

## Figures and Tables

**Table 1:** *E. coli* counts per sampling month in four sites (cfu 100 mL^−1^ water)**.**

Location ID	October-16	November-16	December-16	May-17	June-17	July-17	Mean
Grand Rapids	20	14.3	113	76.2	68.8	128.8	60.7
Miltonville	3230	11.4	140	90.9	69.2	138.9	526.1
Walbridge Park	1440	257	247	140.9	23	446.6	425.7
Docks	1130	56	160	25.1	21.8	196.4	264.9

**Table 2:** Number of *E. coli* isolates (%) expressing resistance to antibiotics, by sampling site, across all sampling events.

Antibiotic	Grand Rapids (n = 69)	Miltonville (n = 78)	Walbridge Park (n = 97)	Docks (n = 85)	Total (n = 329)
Tetracycline	6 (8.7%)	5 (6.4%)	12 (12.3%)	6 (7%)	29 (8.81%)
Imipenem	0 (0%)	0 (0%)	0 (0%)	1 (1.2%)	1 (0.3%)
Cephalothin	59 (85.5%)	72 (92.3%)	92 (94.8%)	81 (95.3%)	309 (95.3%)
Gentamicin	2 (3%)	7 (9%)	9 (9.3%)	9 (10.6%)	27 (8.2%)
Sulfamethoxazole	1 (1.4%)	1 (1.3%)	0 (0%)	3 (3.5%)	5 (1.5%)
Ciprofloxacin	2 (2.9%)	2 (2.5%)	6 (6.2%)	4 (4.7%)	14 (4.2%)
Cefoperazone	3 (4.3%)	2 (2.5%)	2 (2%)	6 (7%)	13 (4%)
Ampicillin	17 (24.6%)	27 (34%)	42 (43.3%)	40 (47%)	126 (38.3%)

**Table 3:** Number of MDR *E. coli* isolates (%) expressing resistance to ≥1, ≥2, ≥3, ≥4 and ≥5 antibiotics, across all sampling events. Total isolate numbers are in parentheses under each sampling site.

MDR Categories	Grand Rapids (n = 69)	Miltonville (n = 78)	Walbridge Park (n = 97)	Docks (n = 85)	Total (n = 329)
≥1 antibiotic	62 (89.8%)	75 (96%)	94 (96.9%)	82 (96.5%)	313 (95%)
≥2 antibiotic	23 (33.3%)	32 (41%)	43 (44.3%)	46 (54.1%)	144 (43%)
≥3 antibiotic	7 (10%)	6 (7.7%)	23 (23.7%)	14 (16.5%)	50 (15%)
≥4 antibiotic	2 (2.9%)	2 (2.5%)	6 (6.2%)	6 (7%)	16 (4.9%)
≥5 antibiotic	1 (1.4%)	1 (1.3%)	0 (0%)	2 (2.3%)	4 (1.2%)

## Data Availability

The data presented in this study are available on request from the corresponding author.
